# Public Perceptions of Gambling in the UK Armed Forces: Understanding Stigma Via a Vignette Experiment

**DOI:** 10.1007/s10899-025-10463-2

**Published:** 2025-12-09

**Authors:** Jessica Smith, Simon Dymond, Jamie Torrance

**Affiliations:** 1 School of Psychology, Swansea University, Singleton Campus, Swansea, SA2 8PP UK; 2 Department of Psychology, Reykjavik University, Reykjavík, Iceland

**Keywords:** Gambling, Harm, Military, Armed forces, Stigma, Public perceptions

## Abstract

Gambling-related harm among armed forces (AF) personnel is a growing concern, yet public perceptions remain underexplored. Anticipated public stigma (the fear of how others perceive you) of gambling among the AF is a potential barrier to help-seeking. Understanding how the public perceives gambling in the AF is essential for shaping stigma-reduction strategies. A randomised, online 2 × 2 between-subjects experiment was conducted. A representative sample of the United Kingdom public (*N* = 396) was recruited through Prolific and randomly assigned to view a vignette featuring either an AF member or non-AF civilian who was described as engaging in either harmful or non-harmful gambling. Following exposure to their assigned vignette, participants completed measures assessing stigma and empathy towards the depicted individual. Participants perceived individuals from the AF as significantly more dangerous (*p* = .002) compared to non-AF civilians. When the vignette depicted gambling-related harm, as opposed to recreational gambling, participants reported significantly higher stigma across seven of the ten stigma measures (all *p* < .001). This study highlights how public stigma towards gambling harm is strong and can be shaped by military status, with AF personnel facing increased perceptions of dangerousness. Findings underscore the need for public stigma-reduction strategies that address both gambling-related and military-specific misconceptions.

## Introduction

Gambling-related harm represents a significant public health concern and is associated with financial instability, strained relationships, and detrimental effects on both physical and psychological health (Muggleton et al., 2021; Wardle et al., 2024). It is also often met with public stigma, considered the general public’s endorsement of negative stereotypes associated with gambling (Hing et al., 2014; Hing et al., 2016b; Horch & Hodgins, 2008; Peter et al., 2019). Within this, stereotypes are operationalised as undesirable and oversimplified characteristics and attributes that are linked to an individual (Link & Phelan, 2001). One of the primary drivers of stigma in gambling is the perception that gambling-related harm is a matter of personal responsibility (Horch & Hodgins, 2008). Language can play a powerful role in shaping these negative stereotypes, as it may encourage judgement and discrimination. For example, the label *‘problem gambler’* often depicts individuals as desperate, aggressive, or lacking self-control (Biggar & Wardle, 2024). Consequently, the stigma surrounding gambling-related harm can significantly hinder treatment-seeking and can perpetuate a lack of societal recognition, as shame and fear often deter individuals from seeking support (Ashubwe & Miano, 2018; Brown & Russell, 2020; Hing, 2015; McGinty et al., 2015). Recent United Kingdom (UK), National Institutes for Health and Care Excellence (NICE) guidelines further emphasise that these barriers may disproportionately impact certain groups (NICE, 2025).

Armed Forces (AF) personnel represent one such potentially stigmatised group, as they often face unique vulnerabilities to gambling-related harm (Champion et al., 2022; Dighton et al., 2023, 2025; Jones et al., 2024; Pritchard & Dymond, 2022). For example, studies have demonstrated higher rates of gambling-related harm among veterans (1.4% vs. 0.2% in the general population) and AF personnel ( 22% vs. 3.8%, respectively) compared with the general population (Dighton et al., 2018); Jones et al., 2024; Public health england, 2023). It has been proposed that the heightened risk of addictive behaviours among AF personnel may be linked to their unique service experiences, including exposure to extreme conditions and traumatic events (Ahern et al., 2015); Pritchard & Dymond, 2022). Additionally, the risk of gambling harm is associated with common demographic and mental health factors observed within the AF population (Jones et al., 2024).

AF personnel already contend with a well-documented and pervasive stigma surrounding mental health issues (Champion et al., 2022; Coleman et al., 2017; Murphy & Busuttil, 2015; Sharp et al., 2015). For example, perceived stigma represents a common barrier to help-seeking for mental health among AF personnel (Champion et al., 2022; Iversen et al., 2011). Perceived stigma is operationalised as the AF personnel’s assumptions about how the public negatively perceives them and subsequently will treat them regarding mental health. For instance, previous research indicates that AF personnel often associate seeking mental health treatment with an increased likelihood of involuntary discharge (Heyman et al., 2022). Research with RAF personnel indicated concerns that they believed the public would see them as less competent because of gambling (Champion et al., 2022). Additionally, perceived stigma surrounding mental health concerns from the public can lead to reluctance to disclose gambling-related concerns due to potential career repercussions in the AF (Champion et al., 2022).

Despite this association, there is little empirical evidence of UK public perceptions held about the AF generally (Hines et al., 2015; Phillips et al., 2023) and it remains unclear how public perceptions will influence stigma related to gambling within the AF. Of the little research there is it indicates that the UK public generally holds AF personnel in high regard, citing respect and admiration (Gribble 2012); Hines et al., 2015). Therefore it remains unclear where AF personnel’s perceptions of public stigma originate, and whether such concerns reflect reality. This warrants particular attention due to the significant role played by the public not only in contributing to stigma but also in influencing recruitment, retention, and support for defence and foreign policy within the military context (Dandeker, 2010; Edmunds, 2012; Gribble (2012). Understanding these public perceptions is also critical for informing effective treatment approaches. Research on veterans highlights that they anticipate judgement from healthcare professionals, especially those without military experience, due to concerns that their experiences will not be fully understood (Coleman et al., 2017). This is particularly important, as much of the existing literature focuses on mental health help-seeking, highlighting a clear gap in research examining help-seeking behaviours related to gambling within military populations (Champion et al., 2022).

Given this gap, attribution theory may offer a valuable framework for understanding public stigma in AF due to its supported application in exploring stigma in mental health and addictive behaviours such as gambling (Correll et al., 2021; Corrigan et al., 2003; Peter et al., 2019). Within this framework, individuals assign attributions for others’ development of mental health concerns, which can foster stigmatising attitudes toward the person (Correll et al., 2021; Corrigan, 2000). Relatedly, there is a critical need to examine how military status influences public perceptions of gambling-related stigma and to investigate how different gambling behaviours shape these perceptions. As to, it enables research to determine the extent to which AF personnel’s concerns are supported by evidence. Furthermore, exploring the role of empathy in shaping attitudes toward gambling in the military is essential, given research suggesting that the public generally holds the military in high regard. Empathy involves recognising and resonating with another person’s emotional distress (Batson et al., 1987, 1997). Understanding these dynamics can inform the development of educational tools and treatment strategies, with clinicians potentially benefiting from integrating approaches that directly address stigma for gambling-related harm in the AF (Coleman et al., 2017).

This study examined how Military status (AF vs. non-AF civilian) influences public perceptions of gambling-related stigma and how gambling behaviour (gambling without harm vs. gambling with harm) shapes these perceptions and explored the role of empathy in attitudes toward military gambling. Gambling with harm was categorised using the clinical criteria for Gambling Disorder identified in the DSM-5 (American Psychological Association, 2013), such as loss of control, continued gambling despite negative consequences, and functional impairment. In contrast, recreational gambling was defined as gambling behaviour that did not meet these criteria and was engaged in gambling without significant adverse effects on personal, financial, or social functioning. Aligning with previous research, the public often perceives military personnel in high regard (Gribble 2012; Hines et al., 2015) but tends to hold strong stigmatising attitudes toward gambling (Hing et al., 2014; Hing et al., 2016b; Horch & Hodgins, 2008; Peter et al., 2019). These perceptions may heighten both negative judgments and sympathetic responses. Additionally, previous research has highlighted that gambling with harm elicits more stigmatising opinions than recreational gambling (Hing et al., 2016b).

Accordingly, we hypothesise the following: First, we hypothesise that members of the public will report higher levels of stigma and empathy when the person engaging in gambling is identified as military rather than non-AF civilian (H1). Second, we hypothesise that gambling behaviour involving harm will elicit greater stigma and empathy than gambling without harm, regardless of military status (H2). Lastly, it is hypothesised that the highest levels of stigma and empathy will be observed when military personnel engage in harmful gambling compared to other combinations of military status and gambling behaviour (e.g., recreational gambling and non-AF civilian or harmful gambling and non- AF civilian)(H3).

## Method

### Design

This randomised online experiment employed a 2 (AF status: Civilian/AF) x 2 (Gambling status: With harm/Without harm) between-subject design. This approach followed recommendations that between-subjects designs in vignette studies can help avoid any sequential effects (Barthels et al., 2025). The experiment was hosted on Qualtrics (Qualtrics, 2023), with the preregistration^1^ and all data/materials^2^ available via the Open Science Framework (OSF). Ethical approval was obtained from (redacted for peer review).

### Participants and Recruitment

**Fig. 1:**
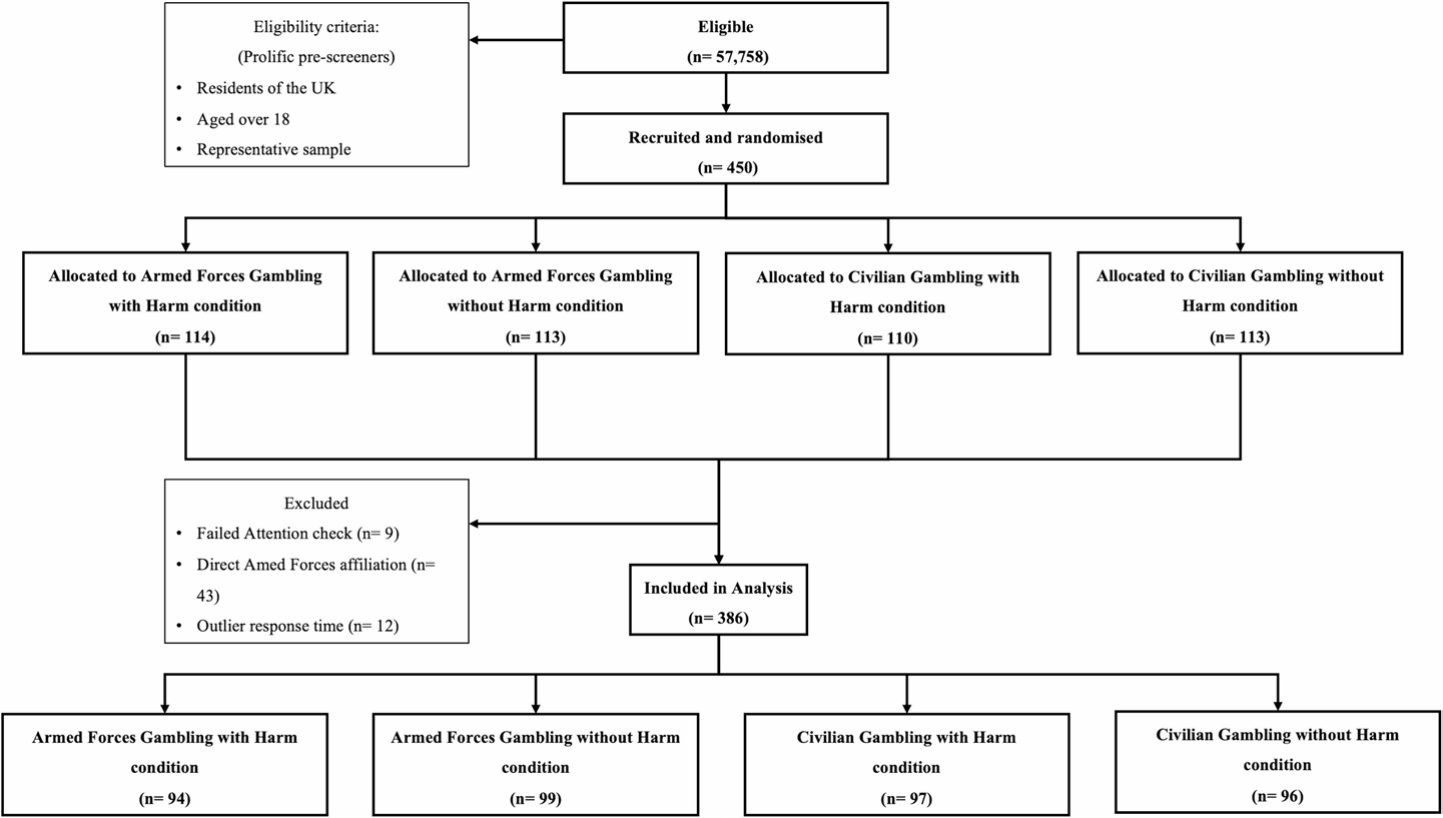
Consolidated Standards of Reporting Trials (CONSORT) flowchart

Participants were recruited via the crowdsourcing platform Prolific and were compensated £0.75 for completing the study (*M*_*Duration*_ = 4.31 min, £10.93 per hour pro rata) (Prolific Academic Ltd., 2023). Only Prolific users who resided in the UK and were aged 18 or over were eligible to participate in the survey. To gauge the unbiased opinions of the UK public, we excluded participants who reported a direct affiliation with the AF. Relatedly, participants were selected using Prolific’s representative sampling function, which reflects the demographic distribution of the UK population. A power analysis indicated a required sample size of 392 participants (power = 0.85, α = 0.05, effect size = 0.15). These parameters were selected based on conventional standards (α = 0.05), a conservative power threshold exceeding the recommended 0.80, and a small-to-medium effect size consistent with prior research in this domain (Correll et al., 2021). To account for potential exclusions due to inattentive or careless responses, we preregistered a target sample size of 450 participants, which was initially achieved. Subsequently, our final sample consisted of 386 participants following the implementation of these exclusions. This is slightly below the pre-specified power analysis estimate of 392. We deemed this minor discrepancy as unlikely to meaningfully affect the results, as the achieved sample size still provided adequate statistical power to detect the expected effects. A Consolidated Standards of Reporting Trials (CONSORT) flowchart is presented in Fig. 1.

### Measures

#### Vignettes

 Each participant was presented with one of four vignettes (Table 1 ). The use of vignettes is an effective and common approach used by researchers to better understand stigmatising perceptions (Erfanian et al., 2020 ; Gourlay et al., 2014 ; McInroy & Beer, 2022 ) and has been repeatedly used in gambling research (Barthels et al., 2025 ; Hing et al., 2016a , b ). The vignettes described a person either experiencing gambling-related harm or engaging in gambling without harm. Additionally, they included a distinguishing factor: whether the individual was a member of the AF or not. The gambling-related harm vignette was developed using the criteria from the Diagnostic and Statistical Manual of Mental Disorders, Fifth Edition (DSM-5; American Psychiatric Association, 2013 ), as well as prior studies that have utilised vignettes to examine gambling behaviours (Peter et al., 2019 ). Before implementation, the vignettes underwent various stages of adaptation by the research team until a consensus was met. To ensure consistency, the ‘gambling without harm’ vignettes were designed to mirror the ‘gambling with harm’ vignettes by maintaining similar sentence structures while adjusting the wording to reflect the absence of harm. In two of the vignettes, the individual was identified as a sergeant and explicitly described as serving in the AF, following the approach of similar studies using AF-related vignettes (O’Donnell et al., 2018 ). A young adult white male of non-commissioned rank (a sergeant) was chosen to match the typical profile of those who engage in gambling-related harm within and outside the AF and to coincide with the overall common demographics of the AF (Gartner et al., 2022 ; Jones et al., 2024 , p. 20; Ministry of Defence, 2025). This was intended to ensure the vignette depicted an individual representative of those most likely to be exposed to stigmatising opinions regarding gambling within the AF. 

**Table 1:** Vignette condition and description

Condition	Vignette text
Gambling with harm + Non-AF civilian	“Mike Jones, a 25-year-old male, enjoys gambling. Recently, the amount of time and money he spends gambling has increased. Gambling has become a central focus in his life, often interfering with important responsibilities. He gambles when he feels distressed. Mike often lies to conceal the extent of his gambling.”
Gambling without harm + Non-AF civilian	“Mike Jones, a 25-year-old male, enjoys gambling. The amount of time and money he spends on gambling has remained the same. Gambling has not become a central focus of his life, and he ensures it doesn’t interfere with his important responsibilities. He does not gamble when he feels distressed. Mike is open and honest about his gambling.”
Gambling with harm + Armed Forces	“Sergeant Mike Jones, a 25-year-old male in the Armed Forces, enjoys gambling. Recently, the amount of time and money he spends gambling has increased. Gambling has become a central focus in his life, often interfering with important responsibilities. He gambles when he feels distressed. Mike often lies to conceal the extent of his gambling.”
Gambling with harm + Armed Forces	“Sergeant Mike Jones, a 25-year-old male in the AF, enjoys gambling. The amount of time and money he spends on gambling has remained the same. Gambling has not become a central focus of his life, and he ensures it doesn’t interfere with his important responsibilities. He does not gamble when he feels distressed. Mike is open and honest about his gambling.”

**Fig. 2:**
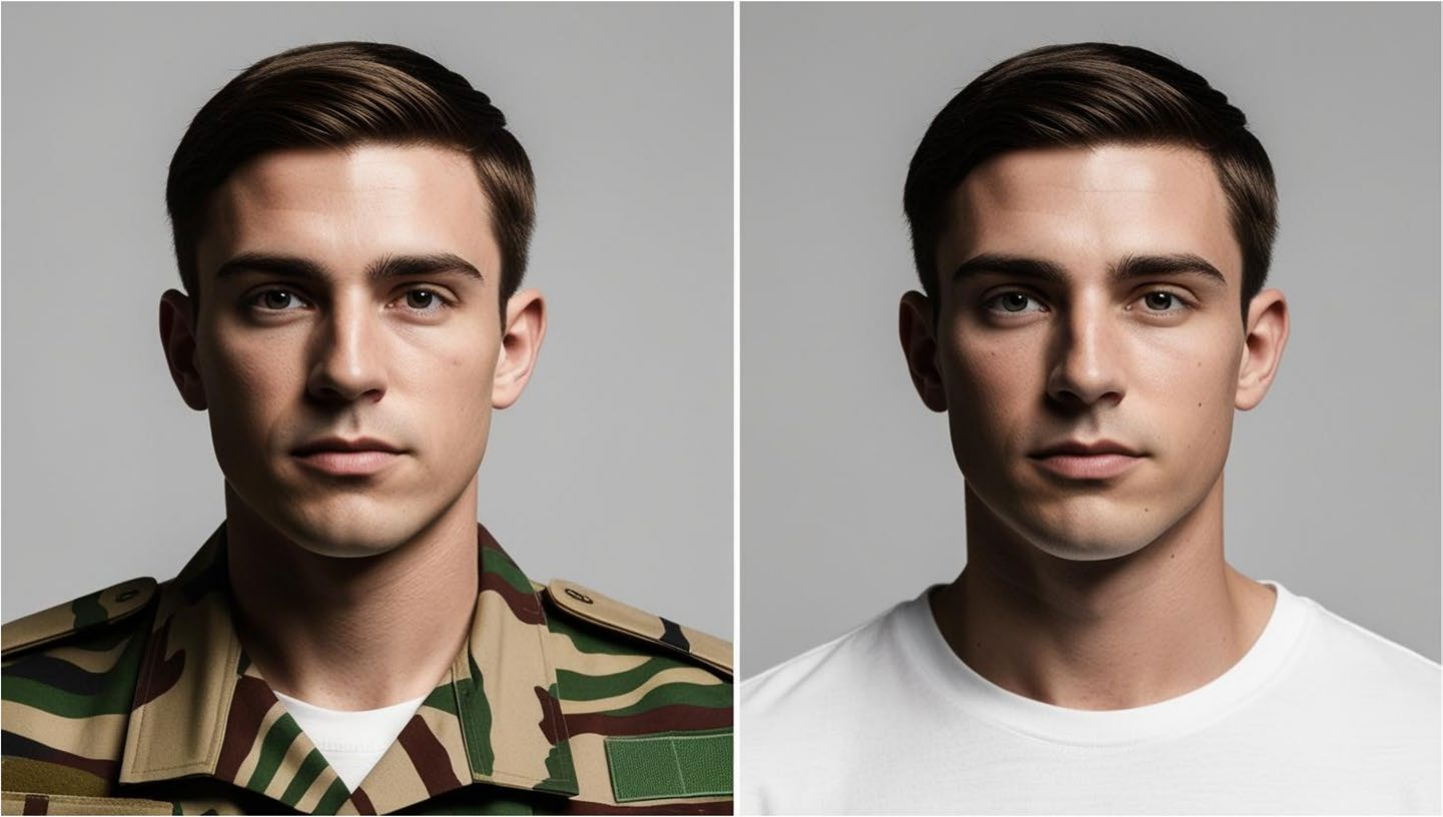
Images used to accompany the vignettes (right: AF, left: civilian)

One of two images accompanied the respective vignettes presented to participants (Fig. 2). The image depicted a young white male dressed either in Non-AF civilian clothing or an AF uniform. The image was adapted using Canva AI, which was instructed to maintain maximal similarity between the images, apart from the clothing (Canva Pty Ltd, 2024). Previous studies have indicated that accompanying images can enhance participant engagement and improve attention to vignettes (Houts et al., 2006; Hughes & Huby, 2004). This approach has also been used in previous vignette-based studies involving military populations (Correll et al., 2021).

#### Stigma

Stigma was measured using the Attribution-9 Questionnaire (AQ-9) (Corrigan et al., 2003), which is widely used for understanding public stigma surrounding mental health concerns and addictive behaviours (Correll et al., 2021; Corrigan et al., 2003; Peter et al., 2019). Via its respective subscales, the AQ-9 measures constructs such as blame (the belief that the individual has control over and is responsible for their mental health concerns), anger (feeling irritated or frustrated due to the individual’s mental health concerns), and pity (feeling sympathy for the individual who is overwhelmed by their mental health concerns). Responses are measured on a 9-point Likert scale of agreement, with higher scores indicative of increased stigmatising opinions. The AQ-9 was developed from the original 27-item Attribution Questionnaire (AQ-27; (Corrigan et al., 2003), which demonstrated good internal consistency (α = 0.70–0.96 across subscales) (Brown, 2008). The AQ-9 comprises the nine items with the highest factor loadings and has also shown acceptable internal consistency in previous research (α = 0.62–0.73) (Corrigan et al., 2003, 2014). In this study Cronbach’s α for the AQ-9 was good (α = 0.83).

#### **Empathy**

 Empathy was measured using the Empathic Concern (EC) subscale of the Empathic Response Questionnaire (Batson et al., 1987 ). This subscale assesses the extent to which six adjectives – sympathetic, warm, moved, soft-hearted, compassionate, and tender, reflect participants’ emotional responses to individuals described in the condition. Participants rated their feelings on a 5-point Likert scale (1 = none at all to 5 = a great deal), indicating the strength of their emotional response. The EC scale has shown strong reliability across various settings and populations, (α = 0.79 to 0.94), within this study the Cronbach’s α of the EC subscale was good (α = 0.80). 

### Procedure

Following recruitment via Prolific and provision of an information sheet, participants consented digitally. Subsequently, participants provided demographic information before being randomly allocated to a condition. Randomisation was conducted using Qualtrics’ built-in randomisation feature, ensuring that each participant was allocated to one of four conditions. One of the four vignettes was subsequently displayed to participants with a mandatory 20-second timer. This measure was implemented to ensure participants did not skip or prematurely advance past the vignette.

After vignette exposure, participants were asked to complete a survey block containing the AQ-9 and EC. To assist with recall, the respective vignette that participants had been exposed to was also displayed at the bottom of the survey block as a reminder. Following the survey block, an attention check was implemented. This attention check required participants to correctly select the name (Mike) used within the vignettes from a set of three options. Participants’ data were excluded if they selected an incorrect name (*n* = 9). Furthermore, as preregistered, we excluded data provided within the 3% fastest response times (*n* = 12) to mitigate careless/rushed responding.

### Statistical Analysis

A multivariate analysis of variance (MANOVA) was conducted to assess (a) the main effect of the AF status condition, (b) the main effect of the gambling status condition, and (c) the interaction effect of AF status and gambling status. If one of the MANOVA effects was statistically significant, follow-up analyses of variance (ANOVAs) were conducted to investigate the effect. The MANOVA included all 11 dependent variables, nine stigma subscales, total stigma, and empathy. We acknowledge that empathy may conceptually overlap with certain stigma subscales; however, including it in the same model enables a comprehensive assessment of participants’ social and emotional responses to the vignettes.

Preliminary screening indicated some non-normality in a few variables due to extreme values. To reduce their influence, these outliers were winsorised, after which the data were suitable for analysis. Winsorising involved replacing extreme outliers with values at the 5th and 95th percentiles (Wilcox, 2003). This process enhances the dataset’s robustness against outliers that do not present meaningful cases. The transformation also improved the analysis by providing a clearer view of the data, leading to more statistically accurate interpretations of the results (Yang et al., 2024).

## Results

The demographic characteristics of the sample were collected as part of the survey and are presented in Table 2. Chi-square tests confirmed that randomisation worked as intended, given that no significant demographic differences (sex, ethnicity, education level, gambling behaviours, and political leaning) were observed between the conditions (all p-values >.116). Participants' self-reported gambling behaviours, both online and in person, are also summarised in Table 2.

**Table 2:** Demographic characteristics and past year gambling engagement split by condition

Attribute	Condition - No. %				
	AF Gambling Harm (*n* = 94)	AF Gambling without Harm (*n* = 99)	Non-AF civilian Gambling Harm (*n* = 97)	Non-AF civilian Gambling without Harm (*n* = 96)	Total (*n* = 386)
Age: Mean (SD)	46.98 (14.27)	46.59 (15.23)	48.97 (16.23)	46.97 (14.80)	47.38 (15)
Sex					
Male	42 (44.1)	49 (49.5)	48 (49.5)	44 (45.8)	183 (47.4)
Female	51 (54.3)	50 (50.5)	49 (50.5)	51 (53.1)	201 (52.1)
Non-binary/third gender	1 (1.1)	0 (0)	0 (0)	1 (1.0)	2 (0.5)
Ethnicity					
White	79 (84)	83 (83.8)	81 (83.5)	83 (86.5)	386 (84.5)
Black	1 (1.1)	4 (4.0)	6 (6.2)	3 (3.1)	14 (3.6)
Asian	8 (8.5)	10 (10.1)	6 (6.2)	9 (9.4)	33 (8.5)
Other	6 (6.4)	2 (2.0)	4 (4.1)	1 (1.0)	13 (3.4)
Education level:					
No formal qualifications	3 (3.2)	1 (1.0)	1 (1.0)	2 (2.1)	7 (1.8)
Secondary education	12 (12.8)	18 (18.2)	24 (24.7)	15 (15.6)	69 (17.9)
Further education	27 (28.7)	18 (18.2)	25 (25.8)	24 (25.0)	94 (24.4)
Undergraduate	29 (30.9)	43 (43.4)	35 (36.1)	34 (35.4	141 (36.5)
Postgraduate degree	19 (20.2)	16 (16.2)	11 (11.3)	18 (18.8)	64 (16.6)
Doctorate	4 (4.3)	3 (3.0)	1 (1.0)	3 (3.1)	11 (2.8)
Past-year gambling engagement					
Online:					
Sports betting	43 (45.7)	34 (34.3)	40 (41.2)	34 (35.7)	151 (39.1)
Slots	20 (21.3)	22 (22.2)	23 (23.7)	13 (13.5)	78 (20.2
Lottery	40 (42.6)	52 (52.2)	48 (49.5)	53 (55.2)	193 (50)
Poker	10 (10.6)	10 (10)	15 (15.5)	5 (5.2)	40 (10.4)
Blackjack	12 (12.8)	11 (11.1)	8 (8.2)	8 (8.3)	39 (10.1)
Roulette	16 (17.0)	13 (13.1)	10 (10.3)	9 (9.4)	48 (12.4)
Scratchcards	20 (21.3)	19 (19.2)	18 (18.6)	13 (13.5)	70 (18.1)
Bingo	17 (18.1)	16 (16.2)	11 (11.3)	12 (12.5)	56 (14.5)
Other	8 (8.5)	5 (5.1)	2 (2)	4 (4.2)	19 (4.9)
In-person/Venue:					
Sports betting	14 (14.9)	15 (15.2)	11 (11)	8 (8.3)	48 (12.4)
Slots	12 (12.8)	16 (16.2)	8 (8.2)	11 (11.5)	47 (12.2)
Lottery	22 (23.4)	25 (25.3)	27 (27.8)	34 (35.4)	108 (28.0)
Poker	8 (8.5)	9 (9.1)	9 (9.3)	5 (5.2)	31 (8.0)
Blackjack	5 (5.3)	10 (10.1)	8 (8.2)	2 (2.1)	25 (6.5)
Roulette	3 (3.2)	9 (9.1)	9 (9.3)	5 (5.2)	26 (6.7)
Scratchcards	22 (23.4)	32 (32.3)	33 (34.0)	35 (36.5)	122 (31.6)
Bingo	17 (18.1)	15 (15.2)	10 (10.3)	13 (13.5)	55 (14.2)
Other	9 (9.6)	7 (7.1)	5 (5.2)	4 (4.2)	25 (6.5)
Political Leaning					
Very liberal/left-wing	8 (8.5)	6 (6.1)	5 (5.2)	10 (10.4)	29 (7.5)
Liberal/left-wing	29 (30.9)	34 (34.3)	37 (38.1)	27 (28.1)	127 (32.9)
Moderate/centrist	33 (35.1)	37 (37.4)	32 (33.0)	39 (40.6)	141 (36.5)
Conservative/right-wing	19 (20.2)	21 (21.2)	20 (20.6)	18 (18.8)	78 (20.2)
Very conservative/right-wing	2 (2.1)	0 (0)	3 (3.1)	1 (1.0)	8 (2.1)
Other	2 (2.1)	0 (0)	0 (0)	0 (0)	3 (0.8)

### Multivariate Analysis

Given violations of Box’s M and Levene’s tests, Pillai’s trace was used as the multivariate test statistic due to its greater robustness to assumption violations (Ateş et al., 2019; Finch, 2005). Assumptions of normality, linearity, and multicollinearity were checked: skewness and kurtosis indicated approximate normality, scatterplots confirmed linear relationships, and correlations showed no multicollinearity issues.

The MANOVA revealed no significant interaction between AF status and gambling status, Pillai’s Trace = 0.03, *F*(3, 372) = 0.968, *p* = .48, η2 = 0.028. This finding indicates that H3, which predicted that participants would provide significantly higher stigma and empathy scores to the AF vignette involving gambling harm compared to all other conditions, was not supported. The lack of a significant interaction effect suggests that the combination of AF status and gambling harm did not produce effects beyond those attributable to each factor independently.

However, AF status had a significant effect upon stigma and empathy scores independently, Pillai’s Trace = 0.06, *F*(1, 372) = 1.99, *p* = .029. This was indicative of a medium effect size (η² = 0.06) and provided partial support for H1 (Co2013). Additionally, gambling status also had a significant effect on stigma and empathy scores independently, Pillai’s Trace = 0.66, hen, *F*(1, 372) = 64.96, *p* < .001. However, unlike AF status, this was indicative of a large effect (η² = 0.66) and strongly supported H2 (Cohen, 1988). Overall scores for each condition can be seen in Table 3.

**Table 3:** Descriptive statistics for stigma and empathy across conditions

Variable	Military gambling with harm M(SD)	Military gambling without harm M(SD)	Non-AF civilian gambling with harm M(SD)	Non-AF civilian gambling without harm M(SD)
Pity	5.99 (0.18)	2.23 (0.17)	5.82 (0.19)	1.97 (0.15)
Danger	4.60 (0.20)	2.19 (0.14)	3.84 (0.19)	1.92 (0.28)
Fear	3.43 (0.19)	1.94 (0.12)	3.19 (0.17)	1.76 (0.11)
Blame	5.31 (0.20)	3.83 (0.25)	5.34 (0.21)	3.79 (0.25)
Segregation	1.95 (0.15)	1.09 (0.04)	1.84 (0.12)	1.25 (0.09)
Anger	3.89 (0.22)	1.63 (0.20)	3.75 (0.19)	1.61 (0.12)
Help	4.98 (0.24)	4.39 (0.23)	4.86 (0.23)	4.20 (0.24)
Avoid	3.65 (0.21)	1.79 (0.15)	3.70 (0.195)	1.72 (0.15)
Coercion	4 (0.22)	1.34 (0.09)	3.34 (0.20)	1.26 (0.08)
Total Stigma	37.86 (0.90)	20.60 (0.73)	35.95 (0.90)	19.71 (0.83)
Empathy	15.45 (0.52)	14.10 (0.48)	15.00 (0.54)	13.53 (0.59)

### Effects of Military Status

Follow-up ANOVAs examining the effect of the AF manipulation on each dependent variable revealed that military personnel were perceived as significantly more dangerous (*M* = 3.36, *SD* = 2.06) compared to non-AF civilians (*M* = 2.88, SD = 1.88), *F*(1, 37) = 9.57, *p* = .002, η² = 0.024. The perceived need to force AF personnel into treatment (coercion) was significantly higher (*M* = 2.64, *SD* = 2.10) compared to non-AF civilians (*M* = 2.31, *SD* = 1.82), *F*(1, 372) = 5.44, *p* = .020, η² = 0.014: however, this effect did not remain significant after applying a Bonferroni correction for multiple comparisons (corrected *p* > .0045). No other significant differences were found between military and non-AF civilian conditions on the remaining variables. These findings also provide only partial support for H1, as higher stigma scores for AF personnel were limited to perceptions of dangerousness, rather than across all stigma dimensions. Additionally, contrary to H1, empathy scores did not significantly differ between military and non-AF civilian conditions.

### Effects of Gambling Status

Separate ANOVAs evaluating the effect of gambling revealed significantly higher stigma ratings across all subscales for vignettes depicting gambling harm compared to those gambling without harm (see Table 4). These results provide strong support for H2. A large effect size was observed for seven of the variables (Pity, Danger, Fear, Anger, Avoidance, Coercion and Stigma Total) emphasising the strength of the relationship between gambling harm and stigma (Cohen, 1988). Participants also reported significantly higher (*p* = .008) empathy toward individuals experiencing gambling-related harm (*M* = 15.22, *SD* = 5.16) compared to those gambling without harm (*M* = 13.82, *SD* = 5.25), represented by a small effect size. Although Empathy and Help-seeking scores were higher for gambling harm, these effects did not remain significant after Bonferroni correction (*p* > .0045), whereas all other variables remained significant.

**Table 4:** Differences in stigma and empathy by gambling status

Variable	Gambling harm		Gambling without Harm			
	Mean	*SD*	Mean	*SD*	*F*	Partial Eta Squared
Pity	5.91	1.80	2.10	1.57	490.95**	0.56
Danger	4.22	1.94	2.05	1.34	166.66**	0.3
Fear	3.31	1.78	1.85	1.13	92.65**	0.2
Blame	5.32	2.00	3.81	2.47	43.52**	0.1
Segregation	1.89	1.32	1.17	0.66	46.40**	0.11
Anger	3.82	2.00	1.62	1.05	183.77**	0.33
Help	4.92	2.27	4.30	2.23	7.109*	0.02
Avoid	3.68	1.98	1.75	1.49	115.55**	0.23
Coercion	3.67	2.07	1.30	0.85	221.13**	0.37
Stigma Total	36.90	8.82	20.16	7.70	396.45**	0.51
Empathy	15.22	5.16	13.82	5.25	7.035*	0.02

*p = *.*008

**p < .001

## Discussion

This study examined how military status and gambling harm influence public perceptions of stigma and empathy. The first hypothesis (H1) proposed that the public would attribute greater levels of stigma and empathy to individuals identified as military personnel compared to non-AF civilians. The second hypothesis (H2) suggested that gambling associated with harm would generate stronger stigma and empathy responses than gambling without harm, irrespective of military status. Finally, it was expected (H3) that the combination of military identity and harmful gambling behaviour would produce the highest stigma and empathy scores relative to all other vignette conditions. Although no significant interaction was found between the two variables, both military status and gambling harm independently had significant effects on participants’ perceptions.

The results regarding gambling-related harm provide strong support for the danger appraisal hypothesis, which suggests that heightened perceptions of danger evoke stronger fear responses and increase the desire for social distance from those perceived as affected (Corrigan et al., 2003; Link et al., 1999). For example, within this study the public viewed Mike gambling with fear, saw him as dangerous and expressed a desire to socially distance from him. This concept is closely linked with attribution theory, which considers how people assign causes to behaviours. Specifically, individuals perceived as dangerous are often seen as more responsible for their condition (Corrigan et al., 2003), highlighting the interplay between perceived threat and causal attributions. Overall findings are consistent with an ongoing narrative that gambling is frequently met with public stigma, often characterised by negative stereotypes (Hing et al., 2014, 2016a, b; Horch & Hodgins, 2008; Peter et al., 2019). Such stigma often discourages individuals from seeking help due to shame and fear of judgement (Ashubwe & Miano, 2018; Brown & Russell, 2020; Hing, 2015; McGinty et al., 2015). The findings suggest that these perceptions are not far-fetched and reflect genuine opinions held by the public.

This stigma surrounding gambling can delay acceptance, as it makes it difficult for individuals to acknowledge they are experiencing gambling-related harm (Hing et al., 2014). This can be explained by the idea that gambling-related harm is sometimes easily concealed, unlike conditions such as Alcohol Use Disorder, which tend to produce more visible symptoms (Brown & Russell, 2020). The hidden nature of gambling harm may therefore contribute to the perception that it is a rare condition, and rare or less visible conditions may often be regarded as ‘deviant’ or ‘abnormal’, which can lead to greater social distance (Hing, 2015; Eldredge et al., 2016). Additionally, this secrecy can contribute to the limited public understanding of how disruptive gambling can be to someone’s life (Brown & Russell, 2020). This can lead the public to respond negatively to the perceived deception involved in concealing gambling problems, which could further intensify stigma (Brown & Russell, 2020). However, this nuanced relationship between secrecy, public perception, and stigma was not fully captured in the current quantitative study and would therefore benefit from further qualitative investigation.

What remained less explored in the previous literature was how this stigma was applied to AF personnel affected by gambling-related harm. In the current study, participants rated AF personnel as more dangerous, by supporting the statement that they perceived mike as dangerous. Such misconceptions may arise from the military’s specialised training, which is often perceived as cultivating aggression and desensitisation (Correll et al., 2021). As a result, military personnel may be viewed as more capable of causing harm than non-AF civilians and, therefore, as inherently more dangerous (Correll et al., 2021). This finding also aligns with the wider “mad, bad, and sad” stereotype, or the “hero, victim, or villain” framing, often debated in public discourse on the Armed Forces (McCartney, 2011; Parry & Pitchford-Hyde, 2023). While not all cited studies use these exact terms, they collectively imply dominant negative portrayals of service members, such as mental vulnerability or moral weakness, rather than empirically supported instability or aggression (Kleykamp & Hipes, 2015; McCartney, 2011; Parrott et al., 2019; Parry & Pitchford-Hyde, 2023; Schmidt, 2020). The media has previously been linked to such stereotypes; in the US, research showed that the news often frames veterans as “charity”, yet simultaneously portrays them as deserving of governmental support and social assistance (Parrott et al., 2019). This dual narrative reinforces the “mad”, “bad”, and “sad” components of the stereotype, casting veterans as both victims of their military experience and as dependents of the state, reinforcing a narrow view of the military veterans (Parrott et al., 2019). While such portrayals may evoke sympathy, they can also perpetuate reductive and stigmatising narratives that overlook the diversity of experiences within the AF, reinforcing assumptions of vulnerability, dysfunction, and dependency. However, limited research has examined how these AF narratives operate within a UK context, particularly regarding gambling.

As to, despite this heightened perception of dangerousness, participants did not report a greater desire for social distance from military personnel, as might be predicted by the danger appraisal hypothesis (B G et al., 1999; Corrigan et al., 2003). This weakens the concept that dangerousness reflects a desire to avoid. Alternatively, this may be linked to the idea that military personnel are held in high regard and subsequently illicit empathy from individuals. Empathy has consistently been linked to prosocial behaviours, including the desire to offer support or assistance (Batson et al., 1981; Paciello, 2013). However, the findings also reflected a notable lack of empathy towards military personnel. Thus, it is of concern if the population are showing reduced empathetic concern towards AF personnel and their struggles with gambling. Military personnel already struggle with help-seeking for mental health due to stigma (Champion et al., 2022; Iversen et al., 2011), and thus the fear of being perceived as dangerous and receiving a non-empathetic approach could further deter them. Therefore, the combination of military-related stereotypes and the stigma surrounding gambling creates a potent narrative, one that may amplify negative perceptions and further marginalise those affected. However, it is cautious to interpret these results as it may be that AQ-9 danger and empathy subscale is not a fully appropriate measure for military populations and may warrant refinement in future research.

Another consideration regarding these results concerns enlisting in the military, unlike previous generations, service members and veterans today typically join the AF by choice rather than through conscription or external obligation. As Correll et al. (2021) state, this voluntary enlistment may lead the public to attribute greater personal responsibility to military personnel for any adverse outcomes associated with their service, viewing it as the result of a chosen occupation. Within this context, attribution theory explains that when a condition is viewed as controllable, it tends to attract greater stigma (Quigley, 2022). For example, one of the primary drivers of stigma in gambling is the perception that gambling-related harm is a matter of personal responsibility (Hing et al., 2016a, b; Horch & Hodgins, 2008). Research has shown that Gambling Disorder is often perceived as less biologically based and more the result of personal failings, such as poor character or lack of self-control, compared to conditions such as Depression or Obsessive–Compulsive Disorder (Hing et al., 2016b; Horch & Hodgins, 2008; Quigley, 2022). These causal attributions have been found to predict stigmatising beliefs and attitudes toward individuals with gambling problems (Barthels et al., 2025; Hing et al., 2016a, b; Horch & Hodgins, 2008). In the present study, such perceptions may have contributed both to the public’s apparent lack of empathy toward military personnel experiencing gambling harm and to broader theories of gambling-related stigma.

### Future Research and Implications

These findings highlight important future research directions and policy implications. Public perceptions of gambling harm suggest a need for education campaigns that frame gambling as a health issue rather than a moral weakness. Such reframing could reduce negative perceptions of military personnel and challenge the endorsement of coercive treatment, as policies grounded in a public health approach can emphasise support and recovery rather than personal blame.

Additionally, future work should investigate how public exposure to veterans and active personnel influences perceptions towards gambling harm. Previous research has identified and implemented several effective stigma-reduction strategies, including contact-based interventions, educational programmes, and advocacy for gambling and mental health (Brown & Russell, 2020; Corrigan et al., 2001, 2012). Adapting such interventions for military settings, particularly through peer-led delivery or leadership endorsement, could foster a more supportive and understanding environment for help-seeking, ultimately shifting negative perceptions and educating the public more on AF life. For example, the *Time to Change* campaign in England, which applied a public health approach through contact-based education and myth-busting, demonstrated a 10% increase in mental health knowledge, a moderate positive shift in attitudes among 13% of people, and a small reduction in the desire for social distance from those with mental distress among 12% of people between 2009 and 2019 (Henderson et al., 2020; Hermaszewska et al., 2022).

Furthermore, qualitative research exploring how individuals construct and justify perceptions towards gambling harm in military personnel, and towards the military overall, particularly through interviews or open-ended surveys, could provide deeper insight into the underlying beliefs that drive stigma and support for coercive intervention.

Finally, these findings may be relevant beyond the UK AF context, with potential implications for other occupational groups exposed to high stress and elevated gambling risk, such as emergency services personnel or healthcare workers. Comparative studies across these populations could help determine whether similar stigma mechanisms are at play.

### Limitations

Several limitations should be acknowledged. Vignettes can lack real-life complexity and nuance (Erfanian et al., 2020). Participants’ responses may not reflect actual social contexts where multiple factors influence decision-making. Our vignettes depicted a white male, which reflects the demographic majority in the AF. However, different results might emerge if we used female or ethnic minority representations instead. Although employing demographically representative sampling, approximately 300 participants’ views cannot definitively reflect wider population perspectives. Finally, given the sensitive nature of stigma and gambling, participants may have adjusted their responses to appear more socially acceptable, particularly on empathy-related items. This social desirability bias, operationalised as the tendency for individuals to present themselves favourably (Holden & Passey, 2009), may have resulted in more positive attitudes being reported than would occur in naturalistic settings. Future research could address this by incorporating strategies to reduce socially desirable responses, such as ensuring greater anonymity or using indirect or implicit measures. More recent recommendations also suggest including a specific indicator that captures the social evaluation of the behaviour and the perceived embarrassment associated with admitting to it (Zhu et al., 2024).

## Conclusion

This study contributes to the growing body of research on public perceptions of gambling harm, with a novel focus on AF personnel. Findings indicate that while the public recognises gambling-related issues, service members are particularly viewed as potentially dangerous, reflecting broader stigma associated with AF stereotypes. Additionally, these perceptions highlight the persistence of negative societal attitudes and misconceptions regarding gambling, underscoring the need for targeted stigma-reduction strategies. Interventions incorporating public education, contact-based approaches, and wider awareness campaigns may help challenge both gambling-related misconceptions and AF-related stereotypes. Future research should continue to explore cultural, media, and interpersonal factors that shape public understanding and responses to gambling harm, particularly in the context of AF service.

## Data Availability

The datasets generated by the survey research during and/or analysed during the current study are available via the Open Science Framework (OSF), https://osf.io/6twha/?view_only=b9e5a8760d5047a9a74a4f33a21927f0.

## Footnotes

1
https://osf.io/6twha/overview?view_only=e44ded7ba2f1454da0cb372be80a0966)

2
https://osf.io/6twha/?view_only=b9e5a8760d5047a9a74a4f33a21927f0.
